# The Prevalence and Molecular Characterization of Bovine Leukemia Virus among Dairy Cattle in Henan Province, China

**DOI:** 10.3390/v16091399

**Published:** 2024-08-31

**Authors:** Yuxi Zhao, Xiaojie Zhu, Zhen Zhang, Jianguo Chen, Yingyu Chen, Changmin Hu, Xi Chen, Ian D. Robertson, Aizhen Guo

**Affiliations:** 1 National Key Laboratory of Agricultural Microbiology, Hubei Hongshan Laboratory, College of Veterinary Medicine, Huazhong Agricultural University, Wuhan 430070, China; yuxiz@webmail.hzau.edu.cn (Y.Z.); xjie0806@163.com (X.Z.); chenjg@mail.hzau.edu.cn (J.C.); chenyingyu@mail.hzau.edu.cn (Y.C.); hcm@mail.hzau.edu.cn (C.H.); chenxi@mail.hzau.edu.cn (X.C.); 2 Hubei International Scientific and Technological Cooperation Base of Veterinary Epidemiology, The Cooperative Innovation Centre for Sustainable Pig Production, Wuhan 430070, China; i.robertson@murdoch.edu.au; 3 School of Veterinary Medicine, Murdoch University, Perth, WA 6150, Australia; 4 Henan Province Seed Industry Development Center, Department of Agriculture and Rural Affairs of Henan Province, Zhengzhou 450045, China

**Keywords:** bovine leukemia virus, prevalence, genotypes, dairy cattle, Bayesian phylodynamic analysis, positive selection

## Abstract

Enzootic bovine leukosis, a neoplastic disease caused by the bovine leukemia virus (BLV), was the primary cancer affecting cattle in China before 1985. Although its prevalence decreased significantly between 1986 and 2000, enzootic bovine leukosis has been re-emerging since 2000. This re-emergence has been largely overlooked, possibly due to the latent nature of BLV infection or the perceived lack of sufficient evidence. This study investigated the molecular epidemiology of BLV infections in dairy cattle in Henan province, Central China. Blood samples from 668 dairy cattle across nine farms were tested using nested polymerase chain reaction assays targeting the partial envelope (*env*) gene (gp51 fragment). Twenty-three samples tested positive (animal-level prevalence of 3.4%; 95% confidence interval: 2.2, 5.1). The full-length *env* gene sequences from these positive samples were obtained and phylogenetically analyzed, along with previously reported sequences from the GenBank database. The sequences from positive samples were clustered into four genotypes (1, 4, 6, and 7). The geographical annotation of the maximum clade credibility trees suggested that the two genotype 1 strains in Henan might have originated from Japan, while the genotype 7 strain is likely to have originated from Moldova. Subsequent Bayesian stochastic search variable selection analysis further indicated a strong geographical association between the Henan strains and Japan, as well as Moldova. The estimated substitution rate for the *env* gene ranged from 4.39 × 10^−4^ to 2.38 × 10^−3^ substitutions per site per year. Additionally, codons 291, 326, 385, and 480 were identified as positively selected sites, potentially associated with membrane fusion, epitope peptide vaccine design, and transmembrane signal transduction. These findings contribute to the broader understanding of BLV epidemiology in Chinese dairy cattle and highlight the need for measures to mitigate further BLV transmission within and between cattle herds in China.

## 1. Introduction

Leukemias, also known as leukoses, are the most common neoplastic diseases in cattle [1]. The clinical presentations of leukemias may include clinical signs, such as anorexia, dyspepsia, decreased milk production, persistent bloating, abomasal displacement, diarrhea, constipation, enlarged superficial lymph nodes, lameness, paralysis, weight loss, generalized weakness or debility, and occasional neurological signs, all of which severely impact the affected animal’s well-being [2].

Bovine leukemia virus (BLV) naturally infects cattle (*Bos taurus taurus* and *Bos taurus indicus*) and *Bubalus bubalis*, and it has been experimentally transmitted to sheep, goats, and alpacas (*Vicugna pacos*) [3]. The primary mode of BLV transmission in nature involves direct exposure to contaminated biological fluids, which might occur during common livestock maintenance tasks (e.g., ear tagging, dehorning, and the reuse of virus-contaminated needles or gloves). Additionally, artificial insemination, embryo transfer, rectal examination, and insect bites have been implicated in BLV transmission [1,3]. Most BLV-infected cattle remain asymptomatic. However, approximately one-third of these cattle exhibit persistent lymphocytosis, characterized by the non-malignant polyclonal expansion of B-cells. A small percentage (1–5%) of infected cattle may develop B-cell leukemia/lymphoma after a prolonged latency period [4]. In addition to reduced milk production, digestive abnormalities, loss of appetite, weight loss, and general weakness, BLV-infected cattle can display multiple neurological symptoms [5].

The BLV genome, composed of 8714 base pairs (bps), contains the essential genes *gag*, *pro*, *pol*, and *env*; the regulatory genes *tax* and *rex*; and the accessory genes *R3* and *G4*. The long terminal repeats are located at the 5′ and 3′ ends [6]. Among the genes in the BLV genome, the *env* gene garners particular attention due to its pivotal role in viral infectivity, immune system interactions, and genetic diversity. Spanning 1548 bps, the *env* gene initially undergoes translation to produce the precursor protein pr72. This precursor is subsequently cleaved by subtilisin/kexin-like convertases, such as furin, into two distinct glycoproteins: gp51 and gp30 [7]. The gp51 protein contains abundant interactive domains and epitopes, including a putative receptor-binding domain [8], conformational and linear antibody epitopes [9], T-cell epitopes [10], and a Zn^2+^-binding domain [11]. The gp30 transmembrane protein is characterized by an N-terminal fusion peptide, which promotes cell fusion and entry [12], as well as a C-terminal cytoplasmic tail, which facilitates viral entry and immune evasion [13]. Unlike gp51, the gp30 protein lacks epitopes and interactive domains; therefore, it exhibits low immunogenic activity. As one of the most important genes in BLV, the *env* gene has received limited attention regarding its nucleotide substitution rate and the pressures of positive selection; however, this aspect is crucial for understanding its role in viral evolution and pathogenicity.

The World Organisation for Animal Health recommends amplifying a 444 bp segment in the gp51 fragment of the partial *env* gene for BLV detection [14]. Based on polymorphisms in this region, 12 genotypes (G1–G12) have been identified [15,16,17,18,19,20]. Among these, the G1 genotype appears to be the most common and widely distributed [21]. However, recent evidence suggests that the 444 bp segment is insufficient for accurate BLV genotyping [22].

A meta-analysis has revealed that BLV is present in 36% of breast cancer samples [23]. This potential association has raised concerns about the public health implications of BLV in humans, prompting further investigations into possible BLV exposures among humans, as well as the potential effects of BLV on human diseases [23,24]. Notably, most BLV-infected cattle remain asymptomatic; relatively few develop lymphoid neoplasms. An in-depth meta-analysis in China found the highest BLV prevalence (38.5%) in cattle sampled prior to 1985 [25]. However, a 2018 study revealed a high BLV prevalence in Chinese dairy cattle, with an animal-level prevalence of 49.1%, highlighting the widespread nature of BLV infection among dairy cattle in China [26].

Although Henan province is a major cattle-producing region in China, there is a lack of systematic investigations regarding BLV epidemiology among dairy cattle within the region, and the circulating genotypes remain unknown. This study aimed to determine the current prevalence of BLV infections among dairy cattle in Henan province; it also aimed to identify specific BLV genotypes circulating within these cattle herds.

## 2. Materials and Methods

### 2.1. Ethical Statement

This study was conducted in accordance with the Australian Code of Practice for the Care and Use of Animals for Scientific Purposes [27]. Approval for this study was obtained from the Animal Ethics Committee and the Human Research Ethics Committee at Murdoch University (permit numbers: R3118/19 and 2019/017), respectively. This study was also approved by the ethics committees overseeing animal and human research at Huazhong Agricultural University.

### 2.2. Sample Collection and DNA Extraction

As of 2021, Henan province contained approximately 364,000 dairy cattle [28]. For this cross-sectional survey, the estimated sample size was calculated as 668, based on an anticipated BLV prevalence of 49.1% [26], a confidence level of 95%, and an allowable error margin of 5% [29].

The nine farms chosen for this study were selected based on convenience; all farms previously cooperated with our organization. These farms are located in seven cities across Henan province: Kaifeng, Zhumadian, Pingdingshan, Nanyang, Jiaozuo, Xinxiang, and Zhengzhou. The sampling locations were chosen to represent four of Henan’s traditional geographical divisions, with the exception of the western region. These divisions included Xinxiang and Jiaozuo, historically significant for dairy farming, and Zhumadian, an emerging center of dairy production. The number of cattle in these herds varied from 440 to 827, with a median of 563; the number of samples collected from each herd was proportional to the herd size.

In total, 668 whole-blood samples (51 calves, 126 heifers, and 491 lactating cows) were collected from clinically healthy cows between June 2021 and May 2022. All cows were raised at their birthplaces and fed using the Total Mixed Ration (TMR) method, all animals were housed in free-stall barns. For each cow, a 1 mL blood sample was extracted from the caudal coccygeal vein, placed in a tube containing ethylenediaminetetraacetic acid as an anticoagulant, and stored at −80 °C until analysis.

Genomic DNA was isolated from 200 μL of each whole-blood sample using the TIANamp Genomic DNA kit (Tiangen Biotech, Beijing, China), in accordance with the manufacturer’s protocol. The extracted DNA (40 μL) was then stored at −20 °C for future use.

### 2.3. Amplification of the env Gene

DNA samples were initially screened using primers specific to the partial *env* gene; samples with positive results were then analyzed with primers specific to the full-length *env* gene. The primers were synthesized by Sangon Biotech (Shanghai, China). Polymerase chain reaction (PCR) amplification was performed in accordance with established procedures described in previous publications, which included the use of specific primers and amplification conditions [8,14]. Detailed primer sequences and reaction conditions are provided in Supplementary File S1. Finally, the PCR products were sequenced by Sangon Biotech (Shanghai, China).

### 2.4. Phylogenetic Analysis of the env Gene

All partial and full-length *env* sequences were edited using Lasergene DNAStar version 7 software (DNAStar, Madison, WI, USA). In total, 383 reference sequences were downloaded from the National Center for Biotechnology Information (NCBI) database [30] as of January 2023, based on the selection criteria of full-length gene sequences (1545–1548 bps), with both sampling time and location provided. Two sequences of the G8 genotype were nearly full-length but not complete. Detailed information on these reference sequences is provided in Supplementary File S2.

For sequence alignment, MAFFT v7.313 software was utilized with the default parameters. The best substitution model was determined using the ModelFinder tool in IQ-TREE v2.2.0 software, based on the Bayesian information criterion. Maximum-likelihood (ML) trees were constructed using the Hasegawa–Kishino–Yano unequal base frequency (i.e., HKY + F) model in IQ-TREE software for the BLV partial and full-length *env* nucleotide sequences. The strengths of the evolutionary relationships were evaluated with nonparametric bootstrap analysis involving 1000 replicates. Sequence alignment (MAFFT v7.313), model testing (ModelFinder), and tree building (IQ-TREE v2.2.0) steps were performed within PhyloSuite v1.2.1 software [31]. Evolutionary divergence distances were calculated using MEGA 7 software [32].

### 2.5. Ancestral State Reconstruction of G1 and G7 in Henan

We employed a Bayesian discrete phylogeographic approach using BEAST v1.10.4 software [33] to reconstruct the ancestral states at key nodes of the phylogenetic tree for country-specific discrete traits. This analysis involved the TN93 + F + G nucleotide substitution model, a relaxed uncorrelated lognormal molecular clock model, and a Bayesian skyline plot tree. Subsequently, maximum clade credibility (MCC) trees were constructed using TreeAnnotator v1.10.4. The trees were then processed for visualization and subjected to graphical editing using FigTree v1.4.3 software.

For each analysis, Bayesian Stochastic Search Variable Selection (BSSVS) was utilized to determine the significance of connections between various countries. Bayes factors (BFs) were computed using SpreaD3 v0.9.7rc software [34]. Connections were considered significant if they had BFs ≥ 3 and a corresponding posterior probability of ≥0.50 for the associated countries.

### 2.6. Rates of Substitution

To calculate the nucleotide substitution rates for different epitopes, we followed the methodology of a previous study [35]. In total, 404 complete *env* sequences were analyzed to classify the critical gene fragments. The substitution rates were assessed using Bayesian Markov chain Monte Carlo methods in BEAST v1.10.4 software. This analysis involved the TN93 + F + G nucleotide substitution model, a relaxed uncorrelated lognormal molecular clock model, and a Bayesian skyline plot tree. The same settings were used when calculating the full-length *env* nucleotide substitution rates for China and other countries.

Each Markov chain Monte Carlo run consisted of 10^8^ steps, with states sampled every 10^4^ steps. Runs were combined using LogCombiner software v1.10.4 (within the BEAST v1.10.4 package). Convergence to the same posterior distribution was assessed using Tracer v1.7.2, which involved evaluating trace plots and ensuring an effective sample size of ≥200 after 10% burn-in [36].

### 2.7. Positive Selection Pressure Analysis

Positive selection analysis of all 404 complete *env* sequences was performed using the DataMonkey Web server suite, with four different methods: fast unconstrained Bayesian approximation for inferring selection (FUBAR), mixed-effects model of episodic selection (MEME), single-likelihood ancestor counting (SLAC), and fixed-effects likelihood (FEL) [37].

### 2.8. Statistical Analysis

Prevalence estimates and corresponding exact binomial 95% confidence intervals (CIs) were calculated using the “binom.test” function in R version 4.3 (R Core Team, Vienna, Austria). Odds ratios (ORs), 95% CIs, and *p*-values (from Fisher’s exact test) were calculated using the “epitools” package in R. The threshold for statistical significance was regarded as *p* < 0.05. ORs and 95% CIs were calculated for farms and cities with at least one positive result.

## 3. Results

### 3.1. Prevalence of BLV

**Figure 1:**
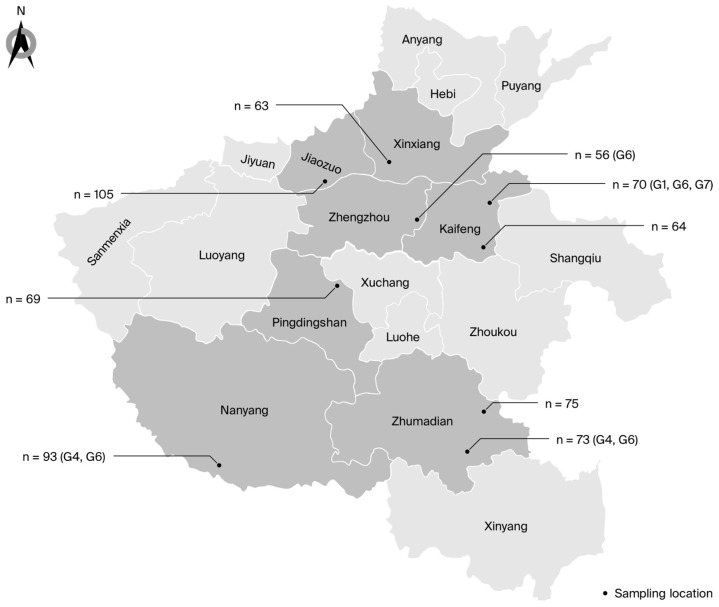
Map showing the distribution, sample sizes, and genotypes of BLV in Henan. “*n*” represents the total number of samples; “Gx” represents the genotypes identified at each farm.

Sample collection locations and quantities are depicted in Figure 1. The PCR amplification of partial *env* gene sequences showed that 23 of 668 dairy cattle tested were BLV-positive, yielding an animal-level prevalence of 3.4% (95% CI: 2.2–5.1) (Table 1). The BLV-positive animals were present on four of the nine farms sampled (farm-level prevalence: 44.4%; 95% CI: 13.7–78.8). The prevalence within infected farms ranged from 1.8% to 11.8% (Table 1 and Table 2). The animals from Farm A were 7.1 (95% CI: 0.9–58.6) times more likely to be BLV-positive (*p* = 0.04) compared to those from Farm I (the farm with the lowest prevalence, used as the reference). Similarly, the OR for BLV in farm F was 7.4 (95% CI: 0.9–58.8) compared with Farm I (*p* = 0.03).

Four of the seven cities sampled contained at least one BLV-positive farm (57.1%; 95% CI: 18.4–90.1). The animal-level prevalence varied among these cities: it was 1.8% in Zhengzhou, 2% in Zhumadian, 6% in Kaifeng, and 11.8% in Nanyang. No positive samples were detected in Jiaozuo, Pingdingshan, or Xinxiang (Table 1 and Table 2). The animal-level prevalence of BLV in Nanyang was 7.4 (95% CI: 0.9–58.8) times higher than that in the reference city, Zhengzhou (*p* = 0.03).

Among calves, heifers, and lactating cows, the lowest prevalence of BLV was observed in calves, with a prevalence of 2.0% (95% CI: 0.05–10.5). The prevalence in heifers was slightly higher, at 2.4% (95% CI: 0.5–6.8), while the highest prevalence was found in lactating cows, at 3.9% (95% CI: 2.3–6.0). Although the risk of BLV infection increased with age, the difference was not statistically significant (*p* > 0.05). The risk of BLV infection in the cows sampled during the spring and summer was higher than that in those sampled during the autumn and winter, with an OR of 3.2 (95% CI: 0.9–10.8). However, this difference was not statistically significant (*p* > 0.05) (Supplementary File S3).

### 3.2. ML-Based Phylogenetic Analysis

**Figure 2:**
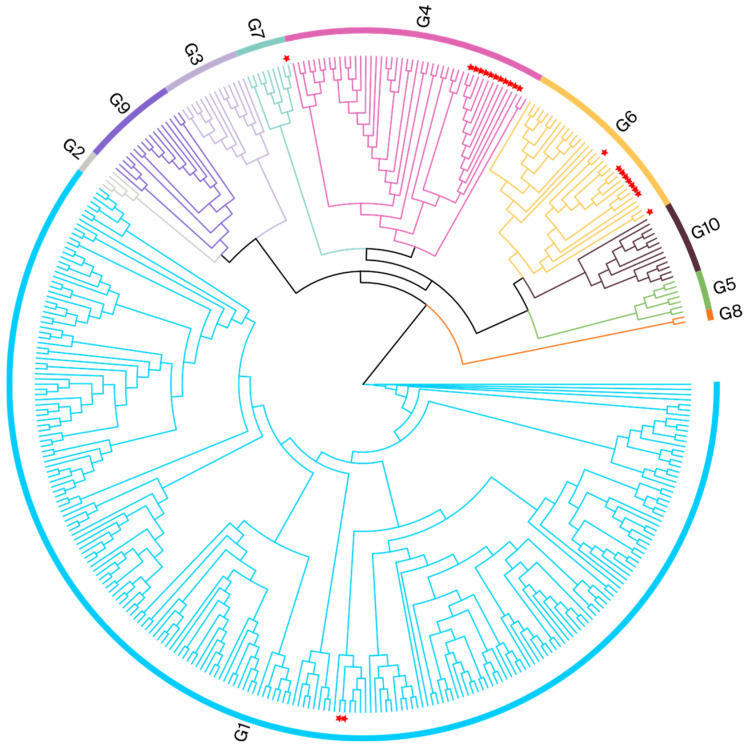
Phylogenetic analysis based on partial *env* gene sequences. BLV isolates from this study are indicated by red pentagrams.

To identify the BLV genotypes circulating in Henan, a phylogenetic analysis was performed using partial *env* gene (370 bps) sequences derived from nested PCR (444 bps fragments). The phylogenetic tree in Figure 2 shows that the 23 samples from Henan were clustered within the G1, G4, G6, and G7 clades. The Henan G1 isolate was related to a Japanese isolate (EF065652), with evolutionary divergences between 0.000 and 0.008. The Henan G4 isolate was clustered with Chinese isolates (MK840877 and MK840879); the evolutionary divergences ranged from 0.003 to 0.011. The Henan G6 isolates were clustered with Vietnamese and Chinese isolates (MH170030, MK840880, and MK840881); the evolutionary divergences were between 0.000 and 0.011. The Henan G7 isolate was clustered with a Russian isolate (JN695881); the evolutionary divergence was 0.005. The gene sequence divergence values are displayed in Supplementary File S4.

**Figure 3:**
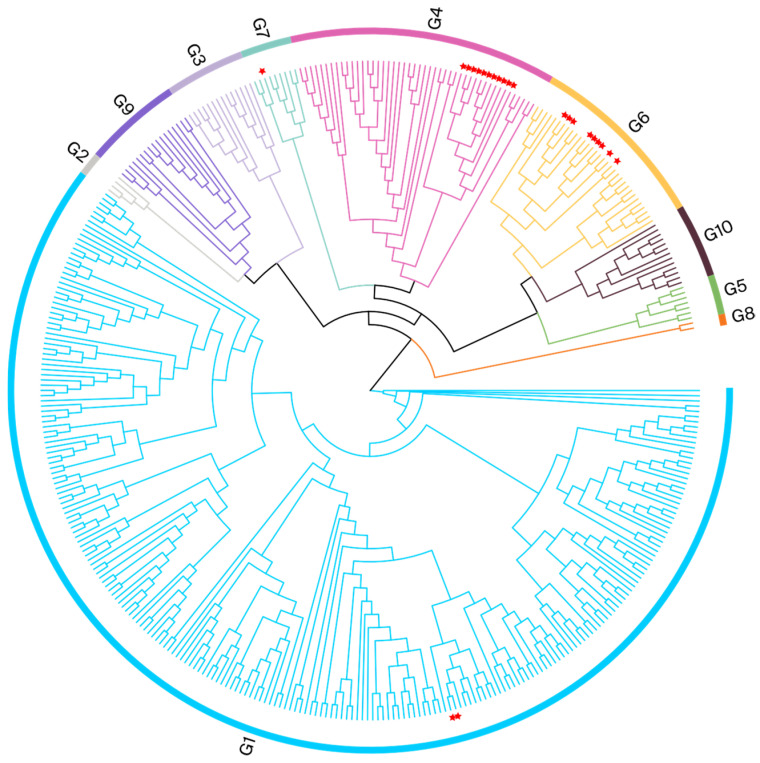
Phylogenetic analysis based on full-length *env* gene sequences. BLV isolates from this study are indicated by red pentagrams.

To validate the findings from phylogenetic analyses conducted using partial env gene sequences and to further explore the evolutionary relationships among isolates from various cities, we selected whole-blood samples from the 23 BLV-positive cows for further analysis. We constructed ML phylogenetic trees based on the alignment of full-length *env* nucleotide sequences (Figure 3).

This validation analysis, using full-length *env* sequences, supported the results of the initial analysis. It demonstrated that Henan isolates belonged to the following genotype groups: G4, twelve; G6, nine; G1, two; and G7, one. The Henan G4 isolate was clustered with Chinese isolates (MK820044 and MK840879); the evolutionary divergences ranged from 0.001 to 0.005. The Henan G6 isolate was also clustered with Chinese isolates (MK840875, MK840878, and MK840880); the evolutionary divergences were between 0.001 and 0.012. The Henan G1 isolate was related to a Vietnamese isolate (MH170027), with evolutionary divergences between 0.003 and 0.004; it was also clustered with Japanese and Mexican isolates on the same branch. The Henan G7 isolate clustered with a Moldovan isolate (KF801458), and the evolutionary divergence was 0.006. The phylogenetic analysis results for the different cities of Henan are presented in Table 3 and Figure 1; the gene sequence divergence values are displayed in Supplementary File S5.

### 3.3. Phylogeographic Reconstruction of Henan G1

The phylogenetic analyses using partial and full-length *env* gene sequences identified two of the isolates as G1. The G1 genotype has been reported in China previously, though only rarely.

**Figure 4:**
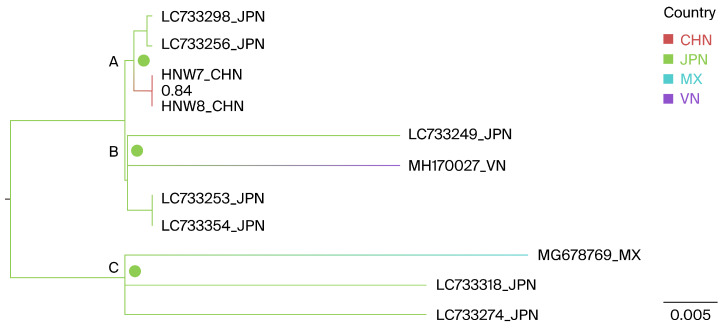
Phylogeographic reconstruction of the BLV G1 genotype. A maximum clade credibility (MCC) tree was constructed using full-length env gene sequences; within the Henan G1 clade, countries were treated as discrete character states. Pie charts at the nodes depict the state posterior probabilities for each country. The labels A–C represent the different notes. Branch colors correspond to the inferred ancestral country with the highest probability, with color transitions representing shifts in the country. CHN, China; JPN, Japan; MX, Mexico; VN, Vietnam.

Geographically annotated MCC trees were used to infer the potential origins of the Henan G1 strains. The results indicate that the two Henan G1 isolates, as well as the Vietnamese MH170027 and the Mexican MG678769, likely originated from Japan, with ancestral state posterior probabilities of 1.0, 0.998, and 1.0, respectively, as shown at nodes A, B, and C in Figure 4.

A BSSVS framework was further utilized to identify the probable geographic locations of transitions between states, focusing on the relationship between the Henan G1 strains and closely related lineages. In the BSSVS analysis, three significant transmission routes for G1 BLV were observed. Japan was identified as having a significant geographic association with the Henan (BFs = 111.6), Mexican (MG678769, BFs = 24.6), and Vietnamese (MH170027, BFs = 24.7) G1 BLV strains, all posterior probabilities > 0.5 (Table 4).

### 3.4. Phylogeographic Reconstruction of Henan G7

**Figure 5:**
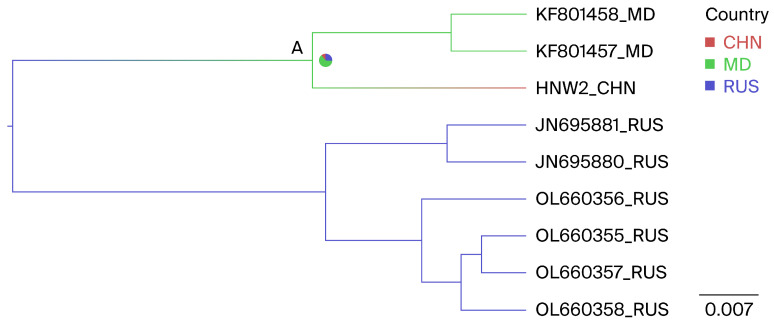
Phylogeographic reconstruction of the BLV G7 genotype. The MCC tree was constructed using full-length *env* gene sequences; within the Henan G7 clade, countries were treated as discrete character states. Pie charts at the nodes depict the state posterior probabilities for each country. The label A represents the note. Branch colors correspond to the inferred ancestral country with the highest probability, with color transitions representing shifts in the country. CHN, China; RUS, Russia; MD, Moldova.

The phylogenetic analyses using partial and full-length *env* gene sequences identified one isolate as G7, a novel genotype within the country. Geographically annotated MCC trees were also used to infer the potential origins of the Henan G7 strain. Figure 5, node A, indicates that the Henan G7 strain may have originated from three countries, including Russia, Moldova, and China, with ancestral state posterior probabilities of 0.11, 0.62, and 0.27, respectively. Given that Moldova has the highest posterior probability, it is inferred that the Henan G7 strain most likely originated from Moldova.

A BSSVS framework was additionally employed to determine the likely geographic locations of state transitions. Two significant transmission routes for G7 BLV were observed. Moldova was identified as having a geographic association with the Chinese G7 BLV strains (BFs Moldova to China = 7.8). Additionally, Russia was identified as having a geographic association with the Moldovan G7 BLV strains, with relatively low supporting evidence linking Russia to Moldova (BFs Russia to Moldova = 3.9), with all posterior probabilities > 0.5 (Table 5).

### 3.5. Nucleotide Substitution Rate Estimation by Bayesian Analysis

Nucleotide substitutions in the full-length *env* gene evolved at a mean rate of 3.15 × 10^−4^ substitutions per site per year (subs/site/year) (95% highest posterior density (HPD): 2.18 × 10^−4^ to 4.30 × 10^−4^ subs/site/year) across 381 sequences. The mean nucleotide substitution rate varied among gene regions, ranging from 4.39 × 10^−4^ subs/site/year to 2.38 × 10^−3^ subs/site/year; the highest rate occurred in the leader segment (2.38 × 10^−4^ subs/site/year), whereas the lowest rate occurred in the gp51 fragment (4.39 × 10^−4^ subs/site/year).

The estimated nucleotide substitution rate in China was 3.93 × 10^−3^ subs/site/year (95% HPD: 6.67 × 10^−4^ to 8.49 × 10^−3^ subs/site/year), over 17 times higher than the rates in other countries (2.25 × 10^−4^ subs/site/year; 95% HPD: 1.63 × 10^−4^ to 2.96 × 10^−4^ subs/site/year) (Table 6).

### 3.6. Positive Selection Sites and Amino Acid Variations in the env Gene

Four methods of selection pressure analysis revealed that four sites within the *env* gene (amino acids 291, 326, 385, and 480) were under positive selection pressure (Table 7). These sites exhibited diversity, and most were hydrophobic. At Site 291, Alanine (A) is the predominant amino acid, with Threonine (T) being replaced by Valine (V) and Glycine (G). At Site 326, Alanine (A) is the majority, with substitutions to Threonine (T), Valine (V), and Serine (S). At Site 385, Proline (P) is the primary amino acid, with substitutions to Leucine (L), Arginine (R), Histidine (H), and Serine (S). Finally, at Site 480, Threonine (T) is the dominant amino acid, with replacements by Alanine (A), Proline (P), Serine (S), and Isoleucine (I). Notably, amino acid changes from Alanine (A), Proline (P), or Threonine (T) to Serine (S) were observed at three sites: 326, 385, and 480 (Table 8).

## 4. Discussion

### 4.1. Prevalence of BLV among Dairy Cattle in Henan

This study revealed substantial differences in the BLV prevalence across farms and cities in Henan province. Farm F had the highest prevalence, at 11.8% (95% CI: 6.1–20.1), whereas Farms G, C, E, B, and H had lower prevalences. At the city level, Nanyang had the highest BLV prevalence of 11.83% (95% CI: 6.1–20.2), whereas Kaifeng, Zhumadian, and Zhengzhou had lower prevalences. Notably, no BLV was detected in cattle from the cities of Pingdingshan, Xinxiang, and Jiaozuo. Variations in BLV prevalence may be influenced by numerous factors, including differences in farm management practices [1,3], geographical conditions [25], and herd size and age [38]. However, further research is needed to fully understand the impacts of these distinct elements on BLV prevalence.

The present study revealed an animal-level BLV prevalence of 3.4% (95% CI: 2.2–5.1), which is higher than the 1.9% previously recorded in Henan [39]. However, this prevalence is lower than the rates observed in other regions of China, including Taiwan (93.6%) [40], the Qinghai–Tibet Plateau (14.8%–19.1%) [41], and Heilongjiang (9.3%) [19]. It is also lower than the rates in countries such as Myanmar (37%) [5], Iran (29.9%) [42], Japan (28.6%) [43], and Thailand (26.2%) [44]. These geographical variations could be attributed to differences among cattle breeds, which might have distinct BLV susceptibilities. Moreover, dissimilar farming practices across regions, including herd management methods, feeding and housing conditions, biosecurity measures, and the frequency of health examinations, could potentially influence BLV prevalence [25,45,46].

### 4.2. Phylogenetic Insights into the BLV env Gene

Phylogenetic analyses provide valuable insights into the global distribution of BLV. Worldwide, three major BLV genotypes (G1, G4, and G6) have been identified. The near-ubiquitous G1 genotype, detected on nearly all continents, has a strong presence in the Americas [35], Europe [47,48], Australia [49], and Asia [45,50]; thus, it is the most prevalent genotype.

The G4 genotype is also widely distributed, particularly in certain European [51,52] and American countries [35]. In Asia, the G4 genotype was initially detected only in Mongolia [17]; however, it has since been identified in other Asian countries [2], including China. Finally, the G6 genotype is mainly prevalent within countries in the Americas [16] and Asia [19,53].

Other genotypes are more geographically restricted: G7 and G8 are found in Eastern Europe and Russia [52]; G2 is found in South American nations [16], as well as Japan (its only Asian location) [54]; G5 is present in Brazil [55] and Costa Rica [35]; G9 occurs in Bolivia [16]; and G10 appears in countries such as China, Myanmar [41,56], and Thailand [44]. Additionally, G11 and G12 are unique to China [19] and Kazakhstan [20], respectively.

In Asia, 11 distinct BLV genotypes have been detected: G1 and G3 in Korea [57,58]; G1 and G6 in the Philippines [2]; G1, G2, and G3 in Japan [35,59]; G1, G6, and G10 in Thailand [44]; G10 in Myanmar [5]; G1, G4, and G7 in Mongolia [17]; G1 and G6 in Jordan [60]; G12 exclusively in Kazakhstan [20]; and G1, G4, G6, G10, and G11 in China [19,41].

In this study, we used both partial and full-length *env* gene sequences to construct ML trees; both methods revealed similar clustering patterns. Based on this analysis, the 23 sequences were separated into four groups: G4, the most prevalent (11/23); G6, with a wide distribution (9/23); G1 (2/23); and G7 (1/23). G1 and G7 were circulating in Kaifeng; G4 was circulating in Zhumadian and Nanyang; and G6 was circulating in Kaifeng, Zhumadian, Nanyang, and Zhengzhou. The current presence of BLV genotypes G1, G4, G6, G7, G10, and G11 places China among the countries with the greatest BLV genotype diversity.

The phylogenetic analysis of full-length *env* gene sequences indicated that all Henan G4 and G6 isolates were related to other Chinese isolates. This genetic similarity suggests a common lineage and geographic origin; the degree of relatedness between these isolates and other Chinese isolates implies local circulation of the virus within the region. Livestock transportation, movement, and trade can facilitate the introduction and dissemination of viruses within and between regions. Inadequate biosecurity measures or handling practices, as well as the mixing of livestock populations, may provide opportunities for enhanced viral circulation.

In this study, a notable finding was that different BLV genotypes were present within a single farm. For example, genotypes 1, 6, and 7 were identified on a farm in Kaifeng, whereas genotypes 4 and 6 coexisted on farms in Zhumadian and Nanyang. The results of previous research suggest that the presence of multiple BLV genotypes within a single host substantially increases the probability of genetic recombination events. These events might lead to new viral strains, with enhanced immune evasion capabilities; they could provide the virus with increased replication potential and better overall fitness, aiding its survival and proliferation [61]. Although the present study did not reveal different BLV genotypes within the same host, it showed that multiple genotypes are prevalent within the same farm, highlighting the importance of long-term monitoring.

### 4.3. Phylogeographic Reconstruction of Henan G1 and G7

In this study, two G1 strains were identified. Although the G1 genotype has been previously reported in China [19], the literature on its origin is minimal. Geographically annotated MCC trees were employed to infer the potential origin of these strains. The analysis suggested that the G1 strains might have originated from Japan. Additionally, the BSSVS results indicated a strong geographic association not only between the Henan G1 isolates and Japanese isolates, but also between the isolates from Vietnam and Mexico. Several factors could be involved in the association with the Japanese strain. First, Japan likely serves as a key supplier of livestock products; it has established close trade relationships with other countries [62]. Livestock trade between countries may increase the likelihood of BLV spillover and transmission between animals, along with the dissemination of BLV. Livestock transportation and movement could create opportunities for BLV spread through improper biosecurity measures or the mixing of animal populations [63].

The G7 genotype, initially identified in Europe and Russia, has since been reported in several Asian countries, as described above, and was also detected in Henan, China. Similarly, geographically annotated MCC trees were used to infer the origin of the Henan G7 strains. The results indicated that the Henan G7 isolates might have originated from Moldova. Further BSSVS analysis also confirmed a geographic association between the Henan isolates and Moldova. Although we have not identified any historical events supporting this conclusion, China has maintained close relationships with some European countries and Russia. Moreover, human migration, animal trade, and foodborne factors (milk and beef) could enable BLV to spread across geographical barriers, hindering prevention efforts [64,65].

Although the geographically annotated MCC trees and BSSVS analyses suggest the potential origins for the Henan G1 and G7 strains, further validation through genetic, epidemiological, and field studies is needed to confirm these findings and understand strain transmission mechanisms. This cross-country transmission underscores the need for international collaboration to monitor, control, and prevent outbreaks. Coordinated global efforts and enhanced data sharing will improve our understanding of the dynamics of cross-border transmission and inform effective BLV control strategies.

### 4.4. BLV env Gene Substitutions and Positive Selection

The phylodynamic analyses showed that the full-length *env* gene substitution rate is 3.15 × 10^−4^ subs/site/year (95% HPD: 2.18 × 10^−4^ to 4.30 × 10^−4^ subs/site/year), which exceeds the *env* gene nucleotide substitution rate (7.84 × 10^−7^ to 2.33 × 10^−5^ subs/site/year) observed for human T-cell lymphotropic virus type-1 (HTLV-I), another member of the *Deltaretrovirus* genus [66]. Although the gp51 fragment of the *env* gene harbors a relatively rich repertoire of epitopes, its nucleotide substitution rate is lower than that of the gp30 fragment, suggesting that these regions have relatively stable sequences. All substitution rates were within the typical range for RNA viruses: 10^−5^ subs/site/year to 10^−2^ subs/site/year [67]. Nevertheless, considering the common inverse relationship between the evolutionary change rate and genome size in microorganisms [68], the substitution rate for the entire BLV genome may be lower than the rate estimated by BEAST, which was solely based on the *env* gene. It is important to acknowledge that fragments and epitopes, with unique biological functions, may change at different rates during viral adaptation to a new environment [69]. Notably, China has a higher rate of nucleotide substitutions in the *env* gene compared to other countries. This variation could be driven by distinct ecological niches and diverse evolutionary histories [70].

Positive selection, the process by which beneficial gene variants are favored over time, is a key driver in the evolution of microorganisms [71]. Our analysis identified four sites (291, 326, 385, and 480) under positive selection, three of which (326, 385, and 480) were located in the gp30 fragment of the *env* gene. This positive selection pressure on the BLV *env* gene may contribute to faster differentiation within the gp30 fragment [72], resulting in a higher nucleotide substitution rate compared to that of the gp51 fragment.

Residue 291 is located at the immunostimulatory A epitope and exhibits positive selection, consistent with previous findings that most amino acid substitutions occur within major epitopes, rather than at random [73]. Virus–cell fusion, mediated by viral membrane glycoproteins, is a crucial step in the infection cycle of all enveloped viruses [74]. BLV transmission mainly occurs through cell–cell fusion because virion instability makes cell-free infection inefficient. Residue 326 is located in the fusion peptide epitope; the fusion peptide destabilizes cellular membranes by oblique insertion into the lipid bilayer, inducing cell fusion [75]. Residue 385 is located in the GD21 epitope, a highly conserved epitope among isolates from different regions; this epitope is found on the surface of *env* subunits, and peptide-derived vaccines are expected to target it [35]. However, variant amino acids should be considered in vaccine development. Residue 480 is located in the cytoplasmic tail motif (CTM) epitope, a 57-amino-acid cytoplasmic domain that contains several motifs, such as dileucine and YXXL motifs, implicated in signaling and membrane protein trafficking. Amino acid variations in residue 480 may impact the ability of the CTM to transmit activation signals and regulate immune recognition [76]. Additionally, amino acid changes in the CTM epitope play key roles in BLV infection. For example, the Y498D mutant provirus is not infectious in sheep [77]. Additionally, Y498A substitution in transfected fetal lamb kidney cells completely prevents the cell–cell propagation of the mutant virus [78]. Previous reports have demonstrated that mutations in certain BLV sites can affect virulence and prevalence [1,79]. Although the overall understanding of the functional outcomes of positive selection pressure at specific sites remains limited, further investigations are warranted.

Although this article may be pioneering in examining the epidemiology and molecular epidemiology of BLV in Henan province, and possibly the first to report the G7 genotype domestically, there are notable limitations to this research. Firstly, the use of a convenience sampling strategy could introduce biases in estimating the prevalence of BLV. To address this, this study increased the sample size. Nevertheless, future research should employ more robust sampling methods to enhance the accuracy of epidemiological estimates. Additionally, this study’s cross-sectional design provides only a snapshot of the BLV situation at a single point in time, which limits the ability to infer temporal trends or causality. Longitudinal studies are required to gain a better understanding of the dynamics of BLV infection over time. Furthermore, the robustness of our dataset, particularly the number and origin of available sequences, may influence some of our results and speculative conclusions. While our analyses are methodologically sound, the limitations in sequence availability could affect the comprehensiveness of our conclusions.

Future research should also consider the inclusion of additional epidemiological factors, such as the age, breed, and management practices of the cattle, which could provide deeper insights into the risk factors associated with BLV infection. Integrating environmental and socio-economic data may further elucidate the broader context of BLV prevalence.

## 5. Conclusions

This study highlights the resurgence of enzootic bovine leukosis in Henan province, China, with a 3.4% animal-level prevalence among dairy cattle. Phylogenetic analysis identified the BLV genotypes 1, 4, 6, and 7, with genotypes 1 and 7 suggesting origins in Japan and Moldova, respectively. The BSSVS analysis confirmed these geographical associations. The estimated substitution rate for the *env* gene indicates ongoing viral evolution, while the positively selected sites identified may be crucial for membrane fusion and vaccine development. These results underscore the need for improved surveillance and control measures to address the re-emergence of BLV and mitigate its spread within and between cattle herds in China.

## Figures and Tables

**Table 1:** Presence of BLV among farms in Henan; * indicates comparison with Farm I in Zhengzhou (lowest non-zero prevalence).

Category	Cities	Herd Size	Samples	nPCR	Animal-Level Prevalence (%)	95% CI (Lower, Upper)	OR	95% CI (Lower, Upper)	*p* *
Farm G	Jiaozuo	827	105	0	0	0–3.5	-	-	-
Farm C	Zhumadian	581	75	0	0	0–4.8	-	-	-
Farm E	Pingdingshan	563	69	0	0	0–5.2	-	-	-
Farm B	Kaifeng	506	64	0	0	0–5.6	-	-	-
Farm H	Xinxiang	498	63	0	0	0–5.7	-	-	-
Farm I	Zhengzhou	440	56	1	1.8	0–9.6	1.0	-	-
Farm D	Zhumadian	574	73	3	4.1	0.9–11.5	2.4	0.2–23.3	0.6
Farm A	Kaifeng	554	70	8	11.4	5.1–21.3	7.1	0.9–58.6	0.04
Farm F	Nanyang	738	93	11	11.8	6.1–20.2	7.4	0.9–58.8	0.03
Total	-	5281	668	23	3.4	2.2–5.1	-	-	-

**Table 2:** Presence of BLV among cities in Henan; * indicates comparison with a city in Zhengzhou (lowest non-zero prevalence).

Cities	Samples	nPCR	Animal-Level Prevalence (%)	95% CI (Lower, Upper)	ORs	95% CI (Lower, Upper)	*p* *
Jiaozuo	105	0	0	0–3.5	-	-	-
Pingdingshan	69	0	0	0–5.2	-	-	-
Xinxiang	63	0	0	0–5.7	-	-	-
Zhengzhou	56	1	1.8	0–9.6	1	-	-
Zhumadian	148	3	2	0.4–5.8	1.1	0.1–11.2	1
Kaifeng	134	8	6	2.6–11.4	3.5	0.4–28.6	0.3
Nanyang	93	11	11.8	6.1–20.1	7.4	0.9–58.8	0.03

**Table 3:** Phylogenetic analysis results across different cities.

Cities	Farm	Partial *env*	Full-Length *env*
Kaifeng	A	G1/G6/G7	G1/G6/G7
Zhumadian	D	G4/G6	G4/G6
Nanyang	F	G4/G6	G4/G6
Zhengzhou	I	G6	G6

**Table 4:** Bayes factors (BFs) in transmission pathways of the G1 genotype.

From	To	BFs	Posterior Probability
Japan	Mexico	24.6	0.92
Japan	Vietnam	24.7	0.92
Japan	China	111.6	0.98

**Table 5:** BFs in transmission pathways of the G7 genotype.

From	To	BFs	Posterior Probability
Moldova	China	7.8	0.86
Russia	China	2.0	0.62
Russia	Moldova	3.9	0.76

**Table 6:** Nucleotide substitution rates of major epitopes. aa—amino acids; HPD—highest posterior density.

Name	aa	Nucleotide	Substitution Rate (Subs/Site/Year)	95% HPD (Subs/Site/Year)
Overall	1–515	1–1545	3.15 × 10^−4^	2.18 × 10^−4^–4.30 × 10^−4^
Leader	1–33	1–99	2.38 × 10^−3^	7.58 × 10^−4^–4.70 × 10^−3^
gp51	34–301	100–903	4.39 × 10^−4^	3.18 × 10^−4^–5.73 × 10^−4^
gp30	302–515	904–1545	5.44 × 10^−4^	3.55 × 10^−4^–7.59 × 10^−4^
China	1–515	1–1545	3.93 × 10^−3^	6.67 × 10^−4^–8.49 × 10^−4^
Others	1–515	1–1545	2.25 × 10^−4^	1.62 × 10^−4^–2.96 × 10^−4^

**Table 7:** Full-length positive selection analysis of *env* gene.

Codon	MEME		FUBAR		SLAC		FEL	
	*β*_+_	*p*	*β*-*α*	Post.Pr	dN-dS	*p*	*α* = *β*	*p*
115	3.08	0.07	0.789	0.83	2.41	0.198	0.852	0.0504
290	15.68	0.05	0.653	0.688	1.65	0.390	1.067	0.2935
291	3.15	0.05	4.513	0.925	5.412	0.054	2.269	0.0379
326	1.96	0.05	2.019	0.921	4.209	0.059	1.965	0.0373
385	2.08	0.05	1.838	0.927	3.603	0.089	1.436	0.0349
479	10,000	0.02	0.071	0.13	0.76	0.527	2.687	0.9392
480	1.81	0.07	1.64	0.898	3.61	0.088	1.281	0.0485
504	25.06	0.04	0.073	0.166	1.201	0.473	2.315	0.9978
505	1062.6	0.01	−0.997	0.131	−2.001	0.872	0.489	0.4844

**Table 8:** Changes of amino acids at positive selection sites.

Site	291			326			385				480			
Majority	A			A			P				T			
Substitution	T	V	G	T	V	S	L	R	H	S	A	P	S	I

## Data Availability

The sequence data generated in this study can be obtained from NCBI, under GenBank accession numbers PP715733–PP715778.

## Supplementary Materials

The following supporting information can be downloaded at: https://www.mdpi.com/article/10.3390/v16091399/s1, Supplementary File S1: Primers and reaction conditions for partial fragment and full-length amplification of *env* gene; Supplementary File S2: 383 reference sequences information; Supplementary File S3: The age group and seasonal distribution of positive samples; Supplementary File S4: Partial *env* gene sequences divergence values; Supplementary File S5: Full-length *env* gene sequences divergence values.

## References

[1] Marawan M.A., Alouffi A., El Tokhy S., Badawy S., Shirani I., Dawood A., Guo A., Almutairi M.M., Alshammari F.A., Selim A. (2021). Bovine leukaemia virus: Current epidemiological circumstance and future prospective. Viruses.

[2] Polat M., Ohno A., Takeshima S.-n., Kim J., Kikuya M., Matsumoto Y., Mingala C.N., Onuma M., Aida Y. (2015). Detection and molecular characterization of bovine leukemia virus in Philippine cattle. Arch. Virol..

[3] Ruiz V., Porta N., Lomónaco M., Trono K., Alvarez I. (2018). Bovine Leukemia Virus Infection in Neonatal Calves. Risk Factors and Control Measures. Front. Vet. Sci..

[4] Gillet N., Florins A., Boxus M., Burteau C., Nigro A., Vandermeers F., Balon H., Bouzar A., Defoiche J., Burny A. (2007). Mechanisms of leukemogenesis induced by bovine leukemia virus: Prospects for novel anti-retroviral therapies in human. Retrovirology.

[5] Polat M., Moe H., Shimogiri T., Moe K., Takeshima S., Aida Y. (2017). The molecular epidemiological study of bovine leukemia virus infection in Myanmar cattle. Arch. Virol..

[6] Ohnuki N., Kobayashi T., Matsuo M., Nishikaku K., Kusama K., Torii Y., Inagaki Y., Hori M., Imakawa K., Satou Y. (2021). A target enrichment high throughput sequencing system for characterization of BLV whole genome sequence, integration sites, clonality and host SNP. Sci. Rep..

[7] Polat M., Takeshima S., Aida Y. (2017). Epidemiology and genetic diversity of bovine leukemia virus. Virol. J..

[8] Suzuki A., Chapman R., Douglass N., Carulei O., van Rensburg J., Williamson A.-L. (2020). Phylogenetic analysis of South African bovine leukaemia virus (BLV) isolates. Viruses.

[9] Pluta A., Albritton L., Rola-Łuszczak M., Kuźmak J. (2018). Computational analysis of envelope glycoproteins from diverse geographical isolates of bovine leukemia virus identifies highly conserved peptide motifs. Retrovirology.

[10] Bai L., Takeshima S., Sato M., Davis W., Wada S., Kohara J., Aida Y. (2019). Mapping of CD4 T-cell epitopes in bovine leukemia virus from five cattle with differential susceptibilities to bovine leukemia virus disease progression. Virol. J..

[11] Gatot J., Callebaut I., Van Lint C., Demonté D., Kerkhofs P., Portetelle D., Burny A., Willems L., Kettmann R. (2002). Bovine leukemia virus SU protein interacts with zinc, and mutations within two interacting regions differently affect viral fusion and infectivity in vivo. J. Virol..

[12] Lorin A., Lins L., Stroobant V., Brasseur R., Charloteaux B. (2007). Determination of the minimal fusion peptide of bovine leukemia virus gp30. Biochem. Biophys. Res. Commun..

[13] Reichert M., Winnicka A., Willems L., Kettmann R., Cantor G. (2001). Role of the proline-rich motif of bovine leukemia virus transmembrane protein gp30 in viral load and pathogenicity in sheep. J. Virol..

[14] Oie A.H.S. (2015). Manual of Diagnostic Tests and Vaccines for Terrestrial Animals.

[15] Asfaw Y., Tsuduku S., Konishi M., Murakami K., Tsuboi T., Wu D., Sentsui H. (2005). Distribution and superinfection of bovine leukemia virus genotypes in Japan. Arch. Virol..

[16] Polat M., Takeshima S., Hosomichi K., Kim J., Miyasaka T., Yamada K., Arainga M., Murakami T., Matsumoto Y., de la Barra Diaz V. (2016). A new genotype of bovine leukemia virus in South America identified by NGS-based whole genome sequencing and molecular evolutionary genetic analysis. Retrovirology.

[17] Ochirkhuu N., Konnai S., Odbileg R., Nishimori A., Okagawa T., Murata S., Ohashi K. (2016). Detection of bovine leukemia virus and identification of its genotype in Mongolian cattle. Arch. Virol..

[18] Moratorio G., Obal G., Dubra A., Correa A., Bianchi S., Buschiazzo A., Cristina J., Pritsch O. (2010). Phylogenetic analysis of bovine leukemia viruses isolated in South America reveals diversification in seven distinct genotypes. Arch. Virol..

[19] Yu C., Wang X., Zhou Y., Wang Y., Zhang X., Zheng Y. (2019). Genotyping bovine leukemia virus in dairy cattle of Heilongjiang, northeastern China. BMC Vet. Res..

[20] Sultanov A., Rola-Łuszczak M., Mamanova S., Ryło A., Osiński Z., Saduakassova M.A., Bashenova E., Kuźmak J. (2022). Molecular characterization of bovine leukemia virus with the evidence of a new genotype circulating in cattle from Kazakhstan. Pathogens.

[21] Lee J., Kim Y., Kang C.S., Cho D.H., Shin D.H., Yum Y.N., Oh J.H., Kim S.H., Hwang M.S., Lim C.J. (2005). Investigation of the bovine leukemia virus proviral DNA in human leukemias and lung cancers in Korea. J. Korean Med. Sci..

[22] Corredor-Figueroa A.P., Salas S., Olaya-Galán N.N., Quintero J.S., Fajardo Á., Soñora M., Moreno P., Cristina J., Sánchez A., Tobón J. (2020). Prevalence and molecular epidemiology of bovine leukemia virus in Colombian cattle. Infect. Genet. Evol..

[23] Gao A., Kouznetsova V., Tsigelny I. (2020). Bovine leukemia virus relation to human breast cancer: Meta-analysis. Microb. Pathog..

[24] Delarmelina E., Buzelin M.A., Souza B.S.D., Souto F.M., Reis J.K.P.D. (2020). High positivity values for bovine leukemia virus in human breast cancer cases from Minas Gerais, Brazil. PLoS ONE.

[25] Ma B., Gong Q., Sheng C., Liu Y., Ge G., Li D., Diao N., Shi K., Li J., Sun Z. (2021). Prevalence of bovine leukemia in 1983-2019 in China: A systematic review and meta-analysis. Microb. Pathog..

[26] Yang Y., Chu S., Shang S., Yang Z., Wang C. (2019). Short communication: Genotyping and single nucleotide polymorphism analysis of bovine leukemia virus in Chinese dairy cattle. J. Dairy Sci..

[27] Hubrecht R. (2013). Revised Australian Code for the care and use of animals for scientific purposes. Anim. Welf..

[28] Department of Agriculture and Rural Affairs of Hainan Province Henan Animal Husbandry Development Achieves a Good Start in 2021. https://www.henan.gov.cn/2021/05-25/2151291.html.

[29] Heller D., Hoppe A., Restrepo S., Gatti L., Tournier A.L., Tapon N., Basler K., Mao Y. (2016). EpiTools: An open-source image analysis toolkit for quantifying epithelial growth dynamics. Dev. Cell.

[30] Sayers E.W., Beck J., Bolton E.E., Bourexis D., Brister J.R., Canese K., Comeau D.C., Funk K., Kim S., Klimke W. (2021). Database resources of the national center for biotechnology information. Nucleic Acids Res..

[31] Zhang D., Gao F., Jakovlić I., Zou H., Zhang J., Li W., Wang G. (2020). PhyloSuite: An integrated and scalable desktop platform for streamlined molecular sequence data management and evolutionary phylogenetics studies. Mol. Ecol. Resour..

[32] Kumar S., Stecher G., Tamura K. (2016). MEGA7: Molecular evolutionary genetics analysis version 7.0 for bigger datasets. Mol. Biol. Evol..

[33] Drummond A.J., Suchard M.A., Xie D., Rambaut A. (2012). Bayesian phylogenetics with BEAUti and the BEAST 1.7. Mol. Biol. Evol..

[34] Bielejec F., Baele G., Vrancken B., Suchard M.A., Rambaut A., Lemey P. (2016). SpreaD3: Interactive visualization of spatiotemporal history and trait evolutionary processes. Mol. Biol. Evol..

[35] Zhao X., Buehring G. (2007). Natural genetic variations in bovine leukemia virus envelope gene: Possible effects of selection and escape. Virology.

[36] Rambaut A., Drummond A.J., Xie D., Baele G., Suchard M.A. (2018). Posterior summarization in Bayesian phylogenetics using Tracer 1.7. Syst. Biol..

[37] Delport W., Poon A.F., Frost S.D., Kosakovsky Pond S.L. (2010). Datamonkey 2010: A suite of phylogenetic analysis tools for evolutionary biology. Bioinformatics.

[38] Kuczewski A., Orsel K., Barkema H., Mason S., Erskine R., van der Meer F. (2021). Invited review: Bovine leukemia virus-Transmission, control, and eradication. J. Dairy Sci..

[39] Yang Y., Fan W., Mao Y., Yang Z., Lu G., Zhang R., Zhang H., Szeto C., Wang C. (2016). Bovine leukemia virus infection in cattle of China: Association with reduced milk production and increased somatic cell score. J. Dairy Sci..

[40] Wang C. (1991). Bovine leukemia virus infection in Taiwan: Epidemiological study. J. Vet. Med. Sci..

[41] Wang M., Wang Y., Baloch A., Pan Y., Xu F., Tian L., Zeng Q. (2018). Molecular epidemiology and characterization of bovine leukemia virus in domestic yaks (*Bos grunniens*) on the Qinghai-Tibet Plateau, China. Arch. Virol..

[42] Mousavi S., Haghparast A., Mohammadi G., Tabatabaeizadeh S. (2014). Prevalence of bovine leukemia virus (BLV) infection in the northeast of Iran. Vet. Res. Forum Int. Q. J..

[43] Murakami K., Kobayashi S., Konishi M., Kameyama K., Tsutsui T. (2013). Nationwide survey of bovine leukemia virus infection among dairy and beef breeding cattle in Japan from 2009–2011. J. Vet. Med. Sci..

[44] Lee E., Kim E.-J., Ratthanophart J., Vitoonpong R., Kim B.-H., Cho I.-S., Song J.-Y., Lee K.-K., Shin Y.-K. (2016). Molecular epidemiological and serological studies of bovine leukemia virus (BLV) infection in Thailand cattle. Infect. Genet. Evol..

[45] Hamada R., Metwally S., Polat M., Borjigin L., Ali A., Abdel-Hady A., Mohamed A., Wada S., Aida Y. (2020). Detection and Molecular Characterization of Bovine Leukemia Virus in Egyptian Dairy Cattle. Front. Vet. Sci..

[46] John E., Keefe G., Cameron M., Stryhn H., McClure J. (2020). Development and implementation of a risk assessment and management program for enzootic bovine leukosis in Atlantic Canada. J. Dairy Sci..

[47] Abbasian B. Molecular Cloning and Sequencing of Bovine Rhinotracheitis Virus GB Gene in Iran. Proceedings of the 3rd Congress of European Microbiological Societies (FEMS 2009).

[48] Rodriguez S.M., Golemba M.D., Campos R.H., Trono K., Jones L.R. (2009). Bovine leukemia virus can be classified into seven genotypes: Evidence for the existence of two novel clades. J. Gen. Virol..

[49] Sherr C., Fedele L., Donner L., Turek L. (1979). Restriction endonuclease mapping of unintegrated proviral DNA of Snyder-Theilen feline sarcoma virus: Localization of sarcoma-specific sequences. J. Virol..

[50] Rola-Łuszczak M., Pluta A., Olech M., Donnik I., Petropavlovskiy M., Gerilovych A., Vinogradova I., Choudhury B., Kuźmak J. (2013). The molecular characterization of bovine leukaemia virus isolates from Eastern Europe and Siberia and its impact on phylogeny. PLoS ONE.

[51] Inabe K., Ikuta K., Aida Y. (1998). Transmission and propagation in cell culture of virus produced by cells transfected with an infectious molecular clone of bovine leukemia virus. Virology.

[52] Matsumura K., Inoue E., Osawa Y., Okazaki K. (2011). Molecular epidemiology of bovine leukemia virus associated with enzootic bovine leukosis in Japan. Virus Res..

[53] Inoue E., Matsumura K., Maekawa K., Nagatsuka K., Nobuta M., Hirata M., Minagawa A., Osawa Y., Okazaki K. (2011). Genetic heterogeneity among bovine leukemia viruses in Japan and their relationship to leukemogenicity. Arch. Virol..

[54] Burgu I., Alkan F., Karaoglu T., Bilge-Dagalp S., Can-Sahna K., Güngör B., Demir B. (2005). Control and eradication programme of enzootic bovine leucosis (EBL) from selected dairy herds in Turkey. DTW. Dtsch. Tierarztl. Wochenschr..

[55] Hemmatzadeh F. (2007). Sequencing and phylogenetic analysis of gp51 gene of bovine leukaemia virus in Iranian isolates. Vet. Res. Commun..

[56] Moe K., Polat M., Borjigin L., Matsuura R., Hein S., Moe H., Aida Y. (2020). New evidence of bovine leukemia virus circulating in Myanmar cattle through epidemiological and molecular characterization. PLoS ONE.

[57] Dube S., Abbott L., Dube D.K., Dolcini G., Gutierrez S., Ceriani C., Juliarena M., Ferrer J., Perzova R., Poiesz B.J. (2009). The complete genomic sequence of an in vivo low replicating BLV strain. Virol. J..

[58] Lee E., Kim E.-J., Joung H.-K., Kim B.-H., Song J.-Y., Cho I.-S., Lee K.-K., Shin Y.-K. (2015). Sequencing and phylogenetic analysis of the gp51 gene from Korean bovine leukemia virus isolates. Virol. J..

[59] Licursi M., Inoshima Y., Wu D., Yokoyama T., González E.T., Sentsui H. (2002). Genetic heterogeneity among bovine leukemia virus genotypes and its relation to humoral responses in hosts. Virus Res..

[60] Ababneh M.M., Al-Rukibat R.K., Hananeh W.M., Nasar A.T., Al-Zghoul M.B. (2012). Detection and molecular characterization of bovine leukemia viruses from Jordan. Arch. Virol..

[61] Yi S., Niu J., Wang H., Dong G., Zhao Y., Dong H., Guo Y., Wang K., Hu G. (2018). Detection and genetic characterization of feline bocavirus in Northeast China. Virol. J..

[62] Sabala E.D., Eric C. (2023). The Impact of Japan’s Trade Agreements and Safeguard Renegotiation on U.S. Access to Japan’s Beef Market.

[63] Gotoh T., Nishimura T., Kuchida K., Mannen H. (2018). The Japanese Wagyu beef industry: Current situation and future prospects—A review. Asian-Australas. J. Anim. Sci..

[64] Schwingel D., Andreolla A.P., Erpen L.M., Frandoloso R., Kreutz L.C. (2019). Bovine leukemia virus DNA associated with breast cancer in women from South Brazil. Sci. Rep..

[65] Pakbin B., Rossen J.W., Brück W.M., Montazeri N., Allahyari S., Dibazar S.P., Abdolvahabi R., Mahmoudi R., Peymani A., Samimi R. (2022). Prevalence of foodborne and zoonotic viral pathogens in raw cow milk samples. FEMS Microbiol. Lett..

[66] Van Dooren S., Pybus O.G., Salemi M., Liu H.-F., Goubau P., Remondegui C., Talarmin A., Gotuzzo E., Alcantara L.C.J., Galvão-Castro B. (2004). The low evolutionary rate of human T-cell lymphotropic virus type-1 confirmed by analysis of vertical transmission chains. Mol. Biol. Evol..

[67] Silva G., Marques N., Nolasco G. (2012). The evolutionary rate of citrus tristeza virus ranks among the rates of the slowest RNA viruses. J. Gen. Virol..

[68] Sanjuán R. (2012). From molecular genetics to phylodynamics: Evolutionary relevance of mutation rates across viruses. PLoS Pathog..

[69] Schuh A.J., Ward M.J., Leigh Brown A.J., Barrett A.D. (2014). Dynamics of the emergence and establishment of a newly dominant genotype of Japanese encephalitis virus throughout Asia. J. Virol..

[70] Yuan J., Wang J., Yu J., Meng F., Zhao Y., Li J., Sun P., Sun S., Zhang Z., Liu C. (2019). Alignment of Rutaceae genomes reveals lower genome fractionation level than Eudicot genomes affected by extra Polyploidization. Front. Plant Sci..

[71] Wu X., Zhou H., Li L., Wang E., Zhou X., Gu Y., Wu X., Shen L., Zeng W. (2020). Whole genome sequencing and comparative genomic analyses of *Lysinibacillus pakistanensis* LZH-9, a halotolerant strain with excellent COD removal capability. Microorganisms.

[72] Renzette N., Gibson L., Bhattacharjee B., Fisher D., Schleiss M.R., Jensen J.D., Kowalik T.F. (2013). Rapid intrahost evolution of human cytomegalovirus is shaped by demography and positive selection. PLoS Genet..

[73] Camargos M.F., Pereda A., Stancek D., Rocha M.A., Reis J.K.P.d., Greiser-Wilke I., Leite R.C. (2007). Molecular characterization of the env gene from Brazilian field isolates of Bovine leukemia virus. Virus Genes.

[74] Maresova L., Pasieka T.J., Grose C. (2001). Varicella-zoster Virus gB and gE coexpression, but not gB or gE alone, leads to abundant fusion and syncytium formation equivalent to those from gH and gL coexpression. J. Virol..

[75] Gatot J.-S., Callebaut I., Mornon J.-P., Portetelle D., Burny A., Kerkhofs P., Kettmann R., Willems L. (1998). Conservative mutations in the immunosuppressive region of the bovine leukemia virus transmembrane protein affect fusion but not infectivity in vivo. J. Biol. Chem..

[76] Novakovic S., Sawai E.T., Radke K. (2004). Dileucine and YXXL motifs in the cytoplasmic tail of the bovine leukemia virus transmembrane envelope protein affect protein expression on the cell surface. J. Virol..

[77] Willems L., Gatot J.-S., Mammerickx M., Portetelle D., Burny A., Kerkhofs P., Kettmann R. (1995). The YXXL signalling motifs of the bovine leukemia virus transmembrane protein are required for in vivo infection and maintenance of high viral loads. J. Virol..

[78] Inabe K., Nishizawa M., Tajima S., Ikuta K., Aida Y. (1999). The YXXL sequences of a transmembrane protein of bovine leukemia virus are required for viral entry and incorporation of viral envelope protein into virions. J. Virol..

[79] Abdala A., Alvarez I., Brossel H., Calvinho L., Carignano H., Franco L., Gazon H., Gillissen C., Hamaidia M., Hoyos C. (2019). BLV: Lessons on vaccine development. Retrovirology.

