# Supplementary material for: The Prevalence and Molecular Characterization of Bovine Leukemia Virus among Dairy Cattle in Henan Province, China
Source: Viruses. 2024 Aug 31;16(9):1399. doi: 10.3390/v16091399 (PMC11437460; doi:10.3390/v16091399)
Supplement: Supplementary file 1 [file viruses-16-01399-s001.zip › Supplementary file S1.pdf]

Primers and corresponding annealing temperatures for amplifying partial env genes

Primer names	Sequences (5'-3')	Annealing temperature (°C)
BLV-env-1	TCTGTGCCAAGTCTCCCAGATA	
BLV-env-2	AACAACAACCTCTGGGAAGGG	58
BLV-env-3	CCCACAAGGGCGGCGCCGGTTT	
BLV-env-4	GCGAGGCCGGGTCCAGAGCTGG	58

The PCR reactions were carried out using two different protocols. The first protocol involved an initial denaturation step at 94°C for 2 minutes, followed by 30 cycles consisting of 30 seconds at 95°C for denaturation, 30 seconds at 58°C for annealing, and 60 seconds at 72°C for extension. The reaction was then subjected to a final extension step at 72°C for 4 minutes. The second PCR reaction was followed the same protocol as the first reaction: 2 minutes at 94°C for initial denaturation, followed by 30 cycles of denaturation at 95°C for 30 seconds, annealing at 58°C for 30 seconds, and extension at 72°C for 60 seconds. A final extension step was conducted at 72°C for 4 minutes [1].

Primers and corresponding annealing temperatures for amplifying env genes in the full-length

Primer names	Sequences (5'-3')	Annealing temperature (°C)
Env Outer Forward	CCTCCTACCAATTCTAAAGACC	
Env Outer Reverse	CACGCAGAAGCGACAATCTC	55
Env Inner Forward	GGGCGGAGAAACACCYAAGG	
Env Inner Reverse	CACTGACTATTCCACTAAGCC	61

The recommended PCR amplification protocol involves an initial denaturation at 95°C for 3 minutes, followed by 25 cycles (or 30 cycles for the second round) consisting of denaturation at 95°C for 15 seconds, annealing at 55°C (or 61°C for the second round) for 15 seconds, extension at 72°C for 2 minutes, and final extension at 72°C for 4 minutes [2].

References

1. Oie, A.H.S. *Manual of Diagnostic Tests and Vaccines for Terrestrial Animals*; Bulletin—Office International des Épizootie: Paris, France, 2015; pp. 1092–1106.
2. Suzuki, A.; Chapman, R.; Douglass, N.; Carulei, O.; van Rensburg, J.; Williamson, A.-L. Phylogenetic analysis of South African bovine leukaemia virus (BLV) isolates. *Viruses* **2020**, *12*, 898.
