# Supplementary material for: The Prevalence and Molecular Characterization of Bovine Leukemia Virus among Dairy Cattle in Henan Province, China
Source: Viruses. 2024 Aug 31;16(9):1399. doi: 10.3390/v16091399 (PMC11437460; doi:10.3390/v16091399)
Supplement: Supplementary file 1 [file viruses-16-01399-s001.zip › Supplementary file S3.pdf]

Table S1 Age group distribution of infected animals

Category	Samples	nPCR	Prevalence (%)	95% CI (Lower, Upper)	OR	95% CI (Lower, Upper)	*p*
calves	51	1	2.0	0.05–10.5	-	-	-
heifers	126	3	2.4	0.5–6.8	1.2	0.1–12.0	1
lactating	491	19	3.9	2.3–6.0	2.0	0.3–15.3	1

Table S2 Seasonal distribution of positive samples

Category (month)	Samples	nPCR	Prevalence (%)	95% CI (Lower, Upper)	OR	95% CI (Lower, Upper)	*p*
Autumn and winter (March to August)	211	3	1.4	0.3–4.1	-	-	-
Spring and Summer (September to February)	457	20	4.4	2.7–6.7	3.2	0.9-10.8	0.07
